# Diffusion-Weighted Mri of Postmenopausal Women with Vaginal Bleeding and Endometrial Thickening: Differentiation of Benign and Malignant Lesions

**DOI:** 10.5334/jbr-btr.1118

**Published:** 2016-07-22

**Authors:** Mehtap Çavuşoğlu, Deniz Sözmen Ciliz, Arzu Ozsoy, Semra Duran, Eda Elverici, Cemal Reşat Atalay, Ozhan Ozdemir, Bulent Sakman

**Affiliations:** 1 Ankara Numune Hospital, TR

**Keywords:** Magnetic resonance imaging, Diffusion-weighted imaging, Endometrial carcinoma, Endometrial polyp, Endometrial hyperplasia

## Abstract

**Purpose:**

To investigate the feasibility of diffusion-weighted magnetic resonance imaging (DWI) with apparent diffusion coefficient (ADC) values in differentiating endometrial cancer from benign endometrial lesions in postmenopausal patients with vaginal bleeding and endometrial thickening and to predict the depth of myometrial invasion in endometrial cancer.

**Materials and Methods:**

Postmenopausal patients with vaginal bleeding and endometrial thickening were enrolled in this prospective study. T2-weighted, pre- and postcontrast T1-weighted and diffusion-weighted images were obtained. The ADC values of all the patients with endometrial pathologies were recorded. The staging accuracies of DWI and postcontrast T1-weighted images in the assessment of myometrial invasion were evaluated in histopathologically proven endometrial cancer patients.

**Results:**

Fifty-two patients (mean age: 57 ± 10, range: 41–79) were enrolled in the study. Thirty-eight of the lesions were benign (27 as hyperplasia and endometritis; 11 as polyps). Fourteen of the 52 endometrial lesions were pathologically proven as cancers and underwent hysterectomy, and all the specimens were reported as endometrioid adenocarcinomas. The mean ADC value (10^–3^ mm^2^/second) of cancer (0.88 ± 0.10) was significantly lower than that of benign lesions (1.78 ± 0.27, p = 0,001). There was no significant difference between ADC values of endometrial tissue in patients with FIGO stage 1A (0.87 ± 0.11, n = 9) and FIGO stage 1B (0.91 ± 0.07, n = 5). The staging accuracy was 92.9 per cent (13/14) for DWI and 85.7 per cent (12/14) for postcontrast T1-weighted images.

**Conclusion:**

ADC values allow benign endometrial lesions to be differentiated from endometrial cancer in postmenopausal patients but do not correlate with the depth of myometrial invasion and histological tumor grading.

## Introduction

Endometrial carcinoma is the most common gynecologic malignancy [1]. It predominantly affects postmenopausal women. Clinically, patients present with abnormal uterine bleeding [2]. Endometrial carcinoma is usually characterized by endometrial thickening or an endometrial mass. Some benign endometrial lesions such as endometrial hyperplasia and endometrial polyps may also cause uterine bleeding and endometrial thickening or a focal mass [3]. Therefore, those pathologies should be discriminated to navigate the treatment process. Transvaginal ultrasonography (TVS) is the most efficient first-step technique for diagnosis of postmenopausal bleeding [4,5]. The sensitivity of TVS to detect endometrial pathologies is high, but its specificity is low [6]. Therefore, biopsy is recommended as a second-step diagnostic method when endometrial thickness exceeds 4 mm [5,6,7,8,9]. However, endometrial biopsy or dilatation and curettage (D&C) may not be possible in postmenopausal patients due to endometrial atrophy, endometrial adhesions, or the requirement of general anesthesia.

Magnetic resonance imaging (MRI), with its superior soft-tissue contrast and multiplanar imaging capability, plays a key role in the evaluation of suspected endometrial pathology [10,11,12]. Kinkel et al. [13] performed a meta-analysis which demonstrated that contrast-enhanced T1-weighted MRI was better than ultrasonography, computerized tomography (CT), or noncontrast MRI. Dynamic contrast-enhanced MRI has been reported to be more accurate in detecting the tumor and the evaluation of myometrial invasion compared to T2-weighted imaging, and this property of dynamic contrast-enhanced MRI was attributed to its much clearer establishment of the border between the tumor and the myometrium [14,15,16]. However, since the risk of development of nephrogenic systemic fibrosis and thus renal failure following contrast-enhanced MRI is known, the necessity of imaging methods without contrast enhancement is progressively increasing in the evaluation of endometrial pathologies and myometrial invasion in patients diagnosed with endometrial cancer. Nevertheless, conventional MRI sequences are not capable of differentiating cancer, endometrial hyperplasia, or benign polyps [17].

Diffusion-weighted MRI (DWI) is a nonenhanced imaging technique that facilitates the display of tissue characteristics based on the difference in diffusion motion of water molecules. Several recent studies have reported DWI to be useful to detect and differentiate endometrial cancer from normal endometrium or a benign lesion [18,19,20]. As endometrial cancer occurs in postmenopausal women with co-morbidities such as obesity, hypertension, and diabetes, it is important to effectively select patients at risk of relapse for more radical surgery and adjuvant treatment. In addition, tumor grade, depth of myometrial invasion, and lymph node status are important for a surgeon to determine the method of treatment, and DWI has also been reported to be beneficial in detecting the depth of myometrial invasion and tumor grade in some recently published studies [21,22,23]. DWI provides the apparent diffusion coefficient (ADC) value of the tissues, which is related to the translational movement of water molecules that is limited in an environment that contains structures such as cell membranes [21]. Although an ADC map obtained from DWI has been reported to be useful to differentiate between normal and cancerous tissue of the endometrium, the diagnostic value of DWI with quantitative analysis of ADC has been controversial, particularly on the depth of myometrial invasion and tumor grade [24,25].

In this study, we aimed to investigate the feasibility of DWI in differentiating endometrial cancer from benign endometrial lesions in postmenopausal patients with vaginal bleeding and endometrial thickening before using invasive diagnostic methods and also to evaluate the myometrial invasion in the endometrial cancer group.

## Material and Methods

### Patient Groups

This prospective study was approved by our Institutional Review Board. Endometrial thickness measurements of less than 4 mm have been reported as appropriate threshold values to exclude endometrial carcinoma in the postmenopausal patients. Any thickness greater than 5 mm in the postmenopausal patients with vaginal bleeding or any endometrial heterogeneity or focal thickening seen at TVS has been regarded as pathological. Informed consent was obtained from all subjects. All patients had undergone magnetic resonance (MR) examinations, including DWI. All lesions were pathologically diagnosed by surgical resection, dilatation, and curettage or transvaginal biopsy.

The exclusion criteria were as follows: (1) patients with any MR contraindication (e.g., cardiac pacemaker, aneurysm clip, pelvic or hip metal prostheses); (2) patients who are claustrophobic or unable to cooperate; (3) patients with any contraindication for contrast material; and (4) patients who were treated at other hospitals. In total, six patients who had complaints of vaginal bleeding and endometrial thickening were not included in the study considering these criteria.

### MRI Protocol

All examinations were performed using a 1.5 Tesla (Optima 450W, General Electrics, Milwaukee, Wisconsin, USA) system with 12-channel body-array torso coils. Both axial and sagittal fast spin-echo T2-weighted images (TR, 4500 ms; TE, 102 ms; matrix size, 384 × 256; FOV, 30 cm; slice thickness, 5.5 mm; gap, 1 mm; number of excitations, 4), both axial and sagittal spin-echo T1-weighted images and gadolinium-enhanced fat-saturated spin-echo T1-weighted images (TR, 550 ms; TE, 6.7 ms; matrix size, 384 × 256; FOV, 32 cm; slice thickness, 5 mm; gap, 1 mm; number of excitations, 2) after the administration of gadolinium dimeglumine (0.1 mmol/l per kg bodyweight) were obtained in all patients. The parameters of both axial and sagittal DWI were as follows: TR, 5500–6000 ms; TE, 76–80 ms; *b* factors 0 and 1000 s/mm^2^; matrix size, 160 × 192; FOV, 30 cm; slice thickness, 5 mm; number of excitations, 8. ADC maps were automatically generated with the manufacturer’s software.

### Image Analysis

The ADC values of benign (endometrial hyperplasia-endometritis and polyps) and malignant endometrial lesions were performed on the ADC maps on the workstation (hp workstation XW 8200). The ADC values were measured in a circular region of interest (ROI) in one representative region as large as possible. Care was taken to exclude necrotic and cystic areas for the ROI on the basis of findings on T2-weighted MR images. When the tumor was not clearly visible on the ADC maps, the ROI was set by referring to T2-weighted images. The measurements were performed by two radiologists with 13 and 12 years of experience in MR imaging. The radiologists were blinded to the histopathological diagnosis. For each case, the ADC values (10^–3^ mm^2^/second) were measured three times in different regions, and the mean measurement values were computed.

Endometrial cancer was diagnosed when the tumor demonstrated high signal intensity on DW images obtained with a b value of 1000 s/mm^2^ and low signal intensity on ADC maps.

DW images are intrinsically T2-weighted images. Therefore, T2 shine-through effect can cause false-positive findings of restricted diffusion if isolated DW images without correlation to ADC maps are used. Tumor was diagnosed when hyperintensity on DW images corresponded to a low ADC value. On the other hand, hyperintensity on DW images that corresponded to a high ADC value was not considered as a tumor tissue.

The staging accuracies of both DWI and postcontrast T1-weighted images in the assessment of myometrial invasion were evaluated in the patients with endometrial cancer. Both radiologists evaluated the lesions separately and then came to a common conclusion together. In order to detect the depth of myometrial invasion, the radiologists assessed the DW images with referring to only T2-weighted echo-planar images (b = 0 s/mm^2^) first. The radiologists assessed the gadolinium-enhanced fat-saturated spin-echo T1-weighted images without referring to other sequences as reported in the study by Takeuchi et al. [20]. Subsequently, these results were compared with the degree of myometrial invasion that was confirmed pathologically. Separate diagnostic accuracy was defined for DWI and postcontrast T1-weighted images.

The depth of myometrial invasion was classified as International Federation of Gynecology and Obstetrics (FIGO) stage 1A (endometrial cancer is limited in the endometrium or invades up to 50% of the myometrium) and FIGO stage 1B (endometrial cancer invades more than 50% of the myometrium).

### Statistical Analysis

Statistical evaluation of the data was performed using SPSS for Windows 11.5 package program. In the evaluations and comparisons which were made according to the benign or malignant nature detected in the pathological examinations, analyses used for the defined variables were the t-test for independent samples and one-way ANOVA, and chi-square test in the evaluation of categorical variables. ROC analysis was used to define the best cut-off value to differentiate malignant and benign lesions for ADC values. A value of P < 0.05 was considered statistically significant.

## Results

From January 2013 to September 2014, 52 postmenopausal patients, aged 41–79 years (mean 57 ± 10 years), with complaints of vaginal bleeding and endometrial thickening (5–46 mm, mean: 16.7 ± 9.3) detected at TVS were enrolled in this study. Thirty-eight (73.1%) of the lesions were benign, and 27 of the 38 benign lesions were pathologically proven as hyperplasia and endometritis; 11 of the 38 benign lesions were pathologically proven as polyps. Fourteen of the 52 (26.9%) endometrial lesions were pathologically proven as cancers. Fourteen of the 52 patients with endometrial lesions underwent hysterectomy from endometrial cancer. All 14 malignant cases were endometrioid adenocarcinomas. The summary of characteristics of patients is given in Table 1.

**Table 1:** Summary of Characteristics of Patients and Histopathology Data.

Characteristics	Patients (n: 52)

Age	57 ± 10 years (41–79 years)
Endometrial thickness	16.7 ± 9.3mm (5–46 mm)
Histology	
Endometritis- hyperplasia	27 (51.9%)
Polip	11 (21.2%)
Endometrioid adenocarcinoma	
Grade 1	10 (19.2%)
Grade 2	4 (7.7%)

**Figure 1:**
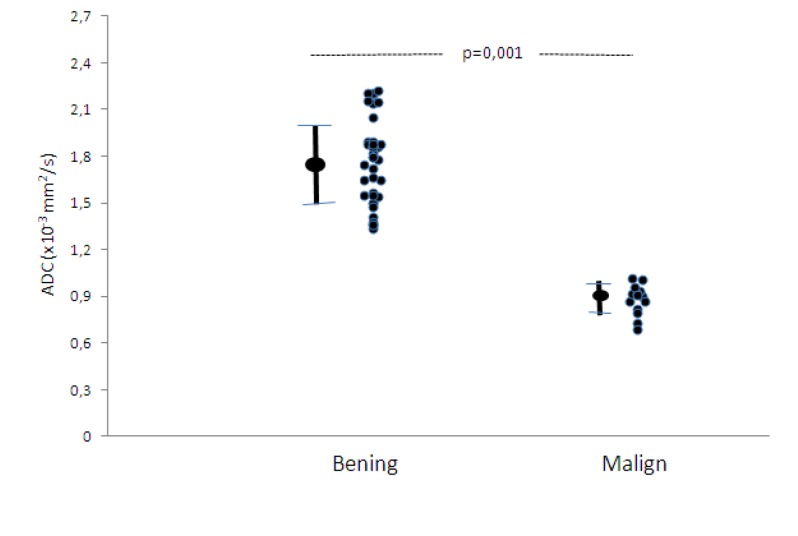
Scatter plots of the ADC values obtained in benign and malignant lesions. The ADC values are significantly different between benign and malignant lesions (P = 0.001).

**Figure 2:**
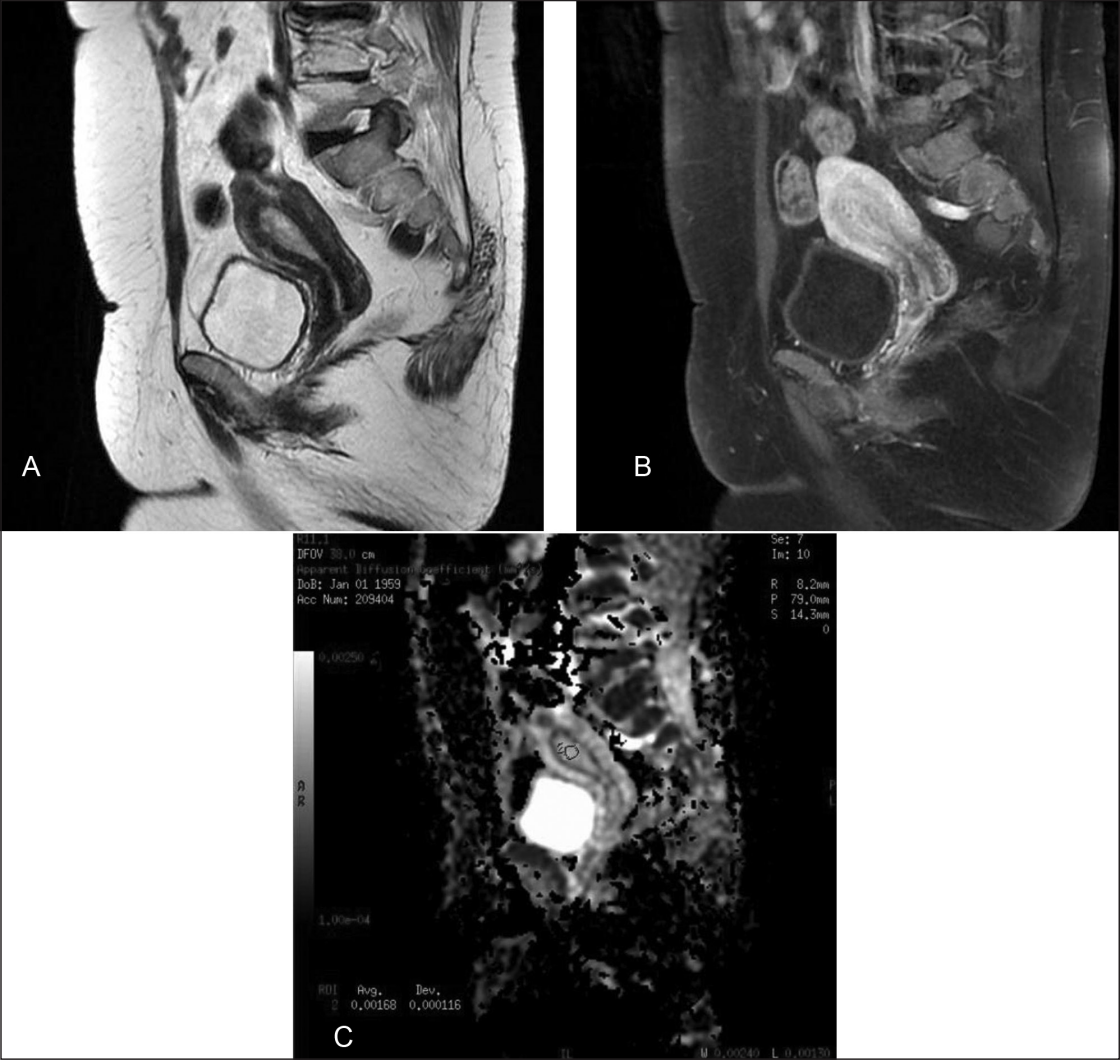
A 54-year-old woman with a histopathologically proven endometrial polyp. Sagittal T2-weighted fast spin-echo image (a) shows a hyperintense mass in the endometrial cavity. Sagittal gadolinium enhanced fat-saturated spin-echo T1-weighted image (b) shows the mass as an enhanced lesion. Sagittal apparent diffusion coefficient (ADC) map image constructed from diffusion-weighted image (b = 1000s/mm^2^) (c). On the ADC map, the tumor shows a high signal intensity, and the ADC value is 1.68x10^–3^ mm^2^/s.

**Figure 3:**
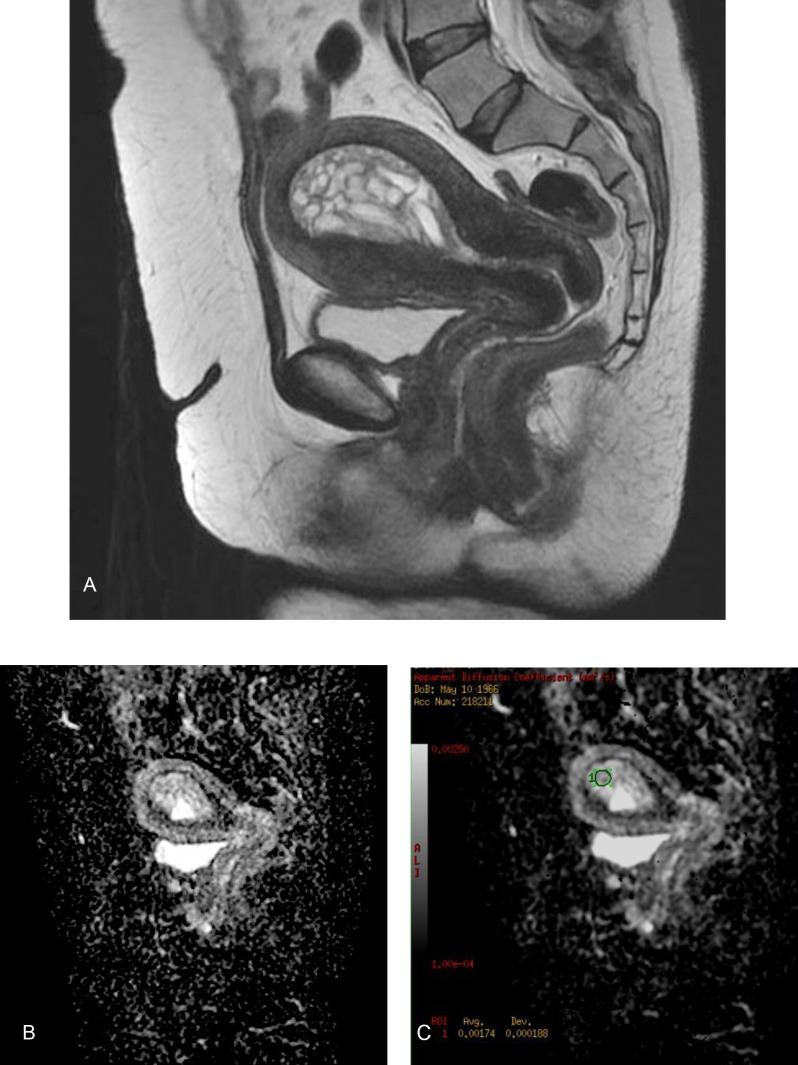
A 47-year-old woman with benign endometrial hyperplasia. Sagittal T2-weighted fast spin-echo image (a) shows diffuse endometrial thickening, and hyperintense cysts are seen in the endometrial cavity. Sagittal ADC map image constructed from diffusion-weighted image (b = 1000s/mm^2^) (b). On the ADC map, the endometrial cavity shows a heterogeneously high signal intensity, and the ADC value is 1.74x10^–3^ (c).

**Figure 4:**
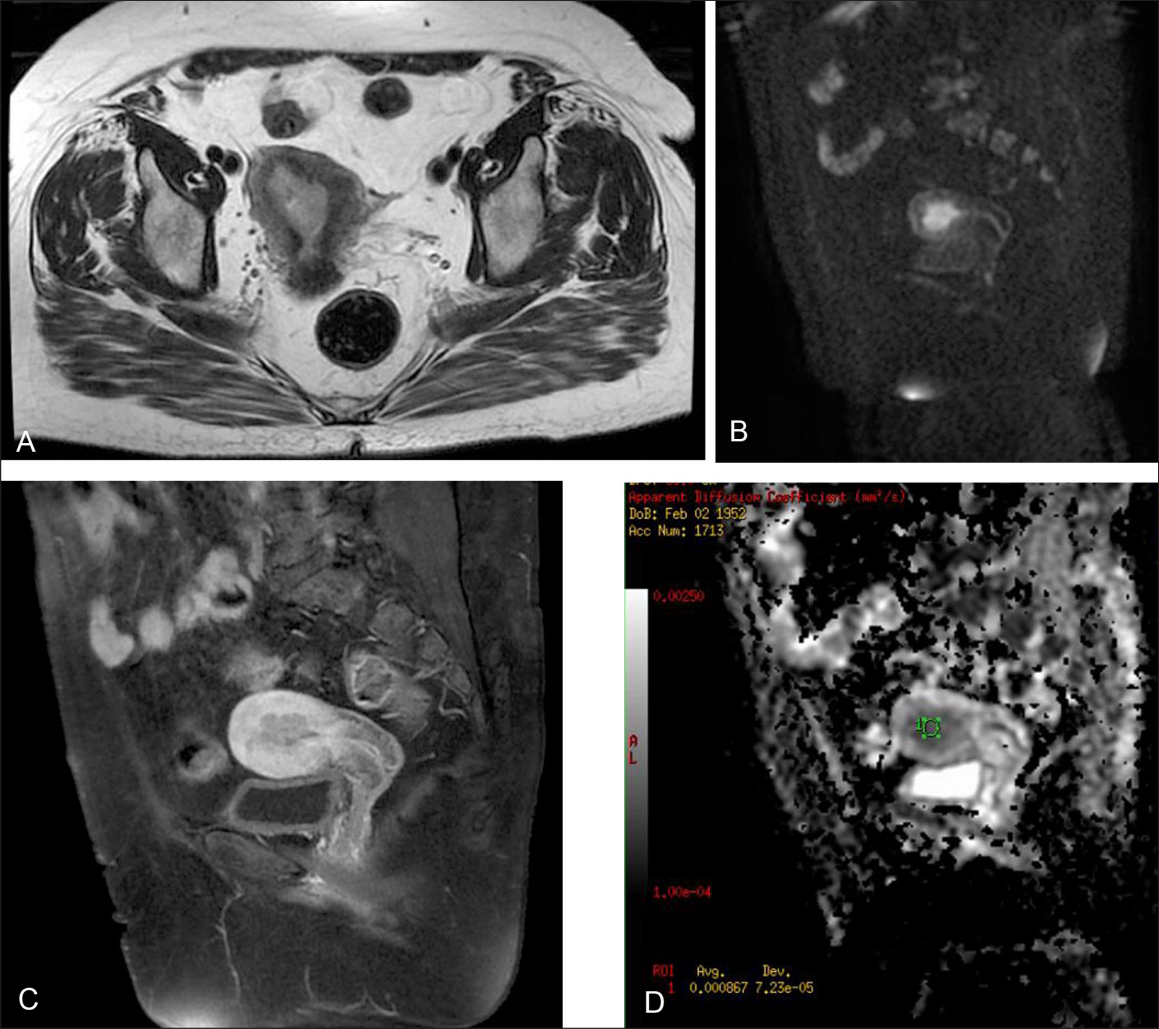
A 48-year-old woman with endometrial cancer. Axial fast spin-echo T2-weighted image shows hyperintense endometrial mass (a). Sagittal diffusion-weighted image (b = 1000s/mm^2^) shows the hyperintense endometrial mass with myometrial invasion (b). Sagittal gadolinium-enhanced fat-saturated spin-echo T1-weighted image shows the tumor as a slightly enhanced lesion (c). Sagittal ADC map image constructed from diffusion-weighted image (b = 1000s/mm^2^). The ADC value is 0.86x10^–3^ (d).

The mean ADC value of endometrial cancer was 0.88 ± 0.10 × 10^–3^ mm^2^/s, and the mean ADC value of benign endometrial lesions was 1.78 ± 0.27 × 10^–3^ mm^2^/s (Figure 1). The ADC values of endometrial carcinoma were significantly lower than those of benign endometrial lesions (P = 0.001) without any overlap with a cut-off value of 1.18 × 10^–3^ mm^2^/s (Figures 2, 3, and 4).

**Figure 5:**
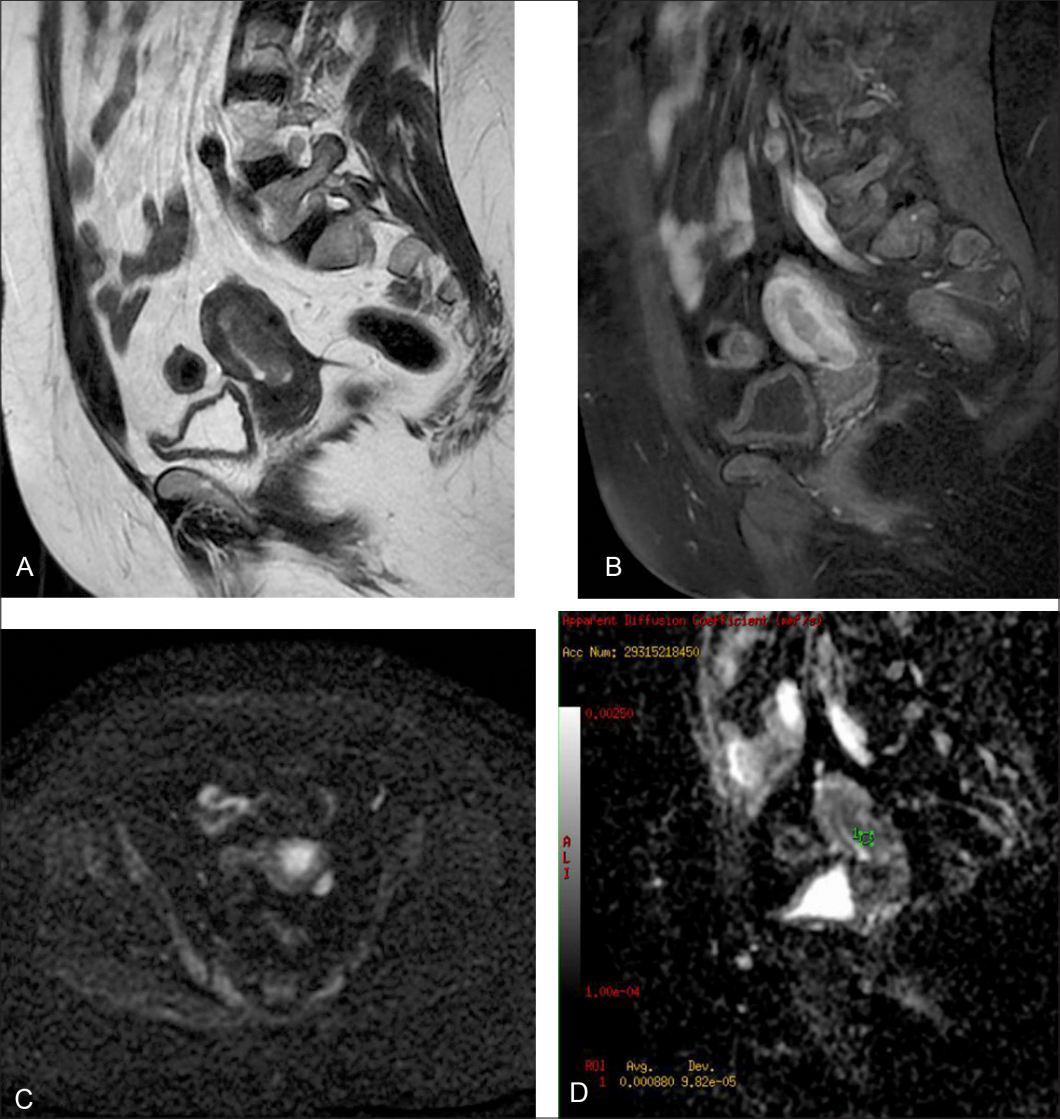
A 56-year-old woman with endometrial cancer. Sagittal T2-weighted fast spin-echo image showing a hyperintense mass in the endometrial cavity (a). Sagittal gadolinium-enhanced fat-saturated spin-echo T1-weighted image shows the tumor as a slightly enhanced lesion (b). Axial diffusion weighted image (b = 1000s/mm^2^) (c) shows hyperintense FIGO stage 1A endometrial mass. The ADC value is 0.88x10^–3^ on the sagittal ADC map image (d).

**Figure 6:**
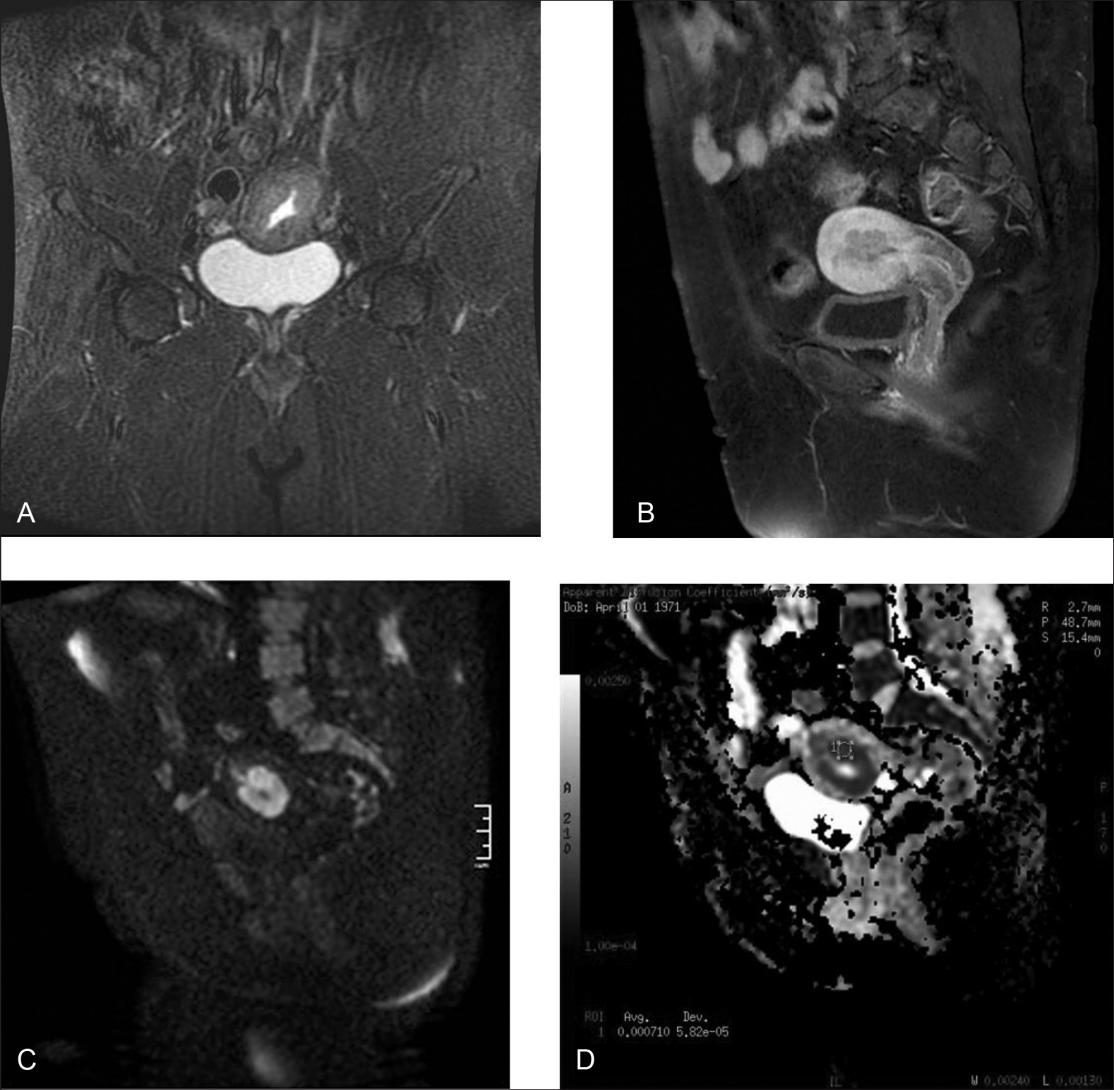
A 42-year-old woman with endometrial cancer. Coronal fast spin-echo T2-weighted image shows the endometrial mass (a). Sagittal gadolinium-enhanced fat-saturated spin-echo T1-weighted image shows the tumor as a slightly enhanced lesion (b). Sagittal diffusion-weighted image (b = 1000s/mm^2^) (c) shows FIGO stage 1B endometrial mass. The ADC value is 0.71x10^–3^ on the sagittal ADC map image (d).

Histological examination of the 14 surgically proven endometrial cancers revealed that 9 of the lesions were confined in the endometrium or invaded up to the inner half of the myometrium (FIGO stage 1A) and that 5 of the lesions invaded the outer half of the myometrium (FIGO stage 1B). The mean ADC value was 0.87 ± 0.11 × 10^–3^ mm^2^/s in patients with FIGO stage 1A and 0.91 ± 0.07 × 10^–3^ mm^2^/s in patients with FIGO stage 1B (Figures 5 and 6). The difference was not statistically significant (p = 0.523).

The staging accuracies of both DWI and postcontrast T1-weighted images in the assessment of myometrial invasion were evaluated in 14 patients with endometrial cancer. The depth of myometrial invasion was underestimated in one lesion on DWI and two lesions on gadolinium-enhanced T1-weighted images. Heterogeneous contrast enhancement of the myometrium made it difficult to detect the tumor margins, resulting in underestimation. The staging accuracy with MRI was 92.9 per cent (13/14) for DWI and 85.7 per cent (12/14) for gadolinium-enhanced T1-weighted images.

In the endometrial cancer group, the mean ADC value for grade 1 tumors (n: 10) was 0.90 ± 0.11 × 10^–3^ mm^2^/s, and the mean ADC value for grade 2 tumors (n: 4) was 0.85 ± 0.05 × 10^–3^ mm^2^/s. The difference was not statistically significant (P = 0.405).

## Discussion

We studied the diagnostic roles of DWI and ADC values in postmenopausal patients with endometrial pathologies and demonstrated that DWI and ADC values provide a diagnostic differentiation between benign and malignant endometrial lesions without any overlapping in these patients. DWI demonstrated the degree of myometrial invasion in endometrial cancer more accurately compared to conventional MRI sequences. On the other hand, it has been established that detection of the degree of myometrial invasion is not possible with ADC values. DWI together with ADC measurement can be used for diagnostic purposes in conditions of difficult endometrial biopsy or curettage due to the presence of endometrial atrophy, adhesions, and the requirement of anesthesia in postmenopausal patients. As endometrial cancer occurs in postmenopausal women with co-morbidities such as obesity, hypertension, and diabetes, DWI is a substantially effective method in detecting the depth of myometrial invasion without causing overtreatment in such patients. Finally, ADC values do not correlate with histological tumor grade.

In the current series, 52 lesions were evaluated at 1.5T with *b* factors 0 and 1000 s/mm^2^. The mean ADC value of endometrial cancer was 0.88 ± 0.10 × 10^–3^ mm^2^/s, and the mean ADC value of benign endometrial lesions (endometrial polyp and hyperplasia) was 1.78 ± 0.27 × 10^–3^ mm^2^/s. The ADC values of endometrial carcinoma were significantly lower than those of benign endometrial lesions without any overlap with a cut-off value of 1.18 × 10^–3^ mm^2^/s. Increased cellularity in endometrial cancer limits the diffusion of water in DWI and lowers the ADC value. On the other hand, benign lesions such as endometrial hyperplasia and endometrial polyps cause an increased ADC value by widening the extracellular area with edematous tissue and cystic components [20]. In the current study, the ADC values in endometrial cancer and benign endometrial lesions were similar to those of Fuji et al. [19], Shen et al. [18], and Takeuchi et al. [20]. Tamai et al. [24] compared the ADC values of 18 women with endometrial carcinomas and 12 women with cervical cancer and pathologically confirmed normal endometrium. The ADC value of endometrial cancer was 0.88(0.16) × 10^–3^ mm^2^/s, which was significantly lower than that of normal endometrium (1.53(0.10) × 10^–3^ mm^2^/s).

The prognostic factors for endometrial cancer are the age of patient, stage, tumor grade and histology, depth of myometrial invasion, cervical involvement, and lymph node metastasis. The detection of preoperative myometrial depth of invasion in endometrial cancer is very important in planning the width of lymph node dissection and predicting the prognosis of the patient. In the latest FIGO classification, a stage IA tumor is limited to the endometrium or invades less than 50 per cent of myometrium, and a stage IB tumor invades equal or more than 50 per cent of myometrium. Pelvic or para-aortic lymph node metastasis increases six to seven fold in deep myometrial invasion [26]. The current standard surgical procedure is total hysterectomy, bilateral salpingo-oophorectomy, and peritoneal cytology. Lymph node dissection is planned in patients with more than 50 per cent myometrial invasion, cervical extension, or extra-uterine disease since the risk of nodal metastasis is high in those patients. However, since the incidence of systemic diseases such as obesity, hypertension, and diabetes is high in postmenopausal patients, overtreatment should be avoided in low-risk patients. Therefore, detecting the degree of myometrial invasion is very important in planning the treatment.

Endometrial carcinoma typically demonstrates diffusion restriction in DWI, which is observed as hyperintense compared to surrounding myometrium and has a hypointense signal property in ADC mapping. When used together with T2-weighted imaging, DWI is a highly accurate technique for assessing the invasion of the deep myometrium. In this context, the diagnosis performance of the DWI technique was equal to or slightly higher than that of contrast-enhanced MRI [18,20,21,27]. Recently, Bonatti et al. [28] compared the diagnostic performance of T2-weighted images plus contrast-enhanced T1-weighted images with the one of T2-weighted images plus DWI in the assessment of myometrial infiltration by endometrial carcinoma, and they reported that T2-weighted images plus DWI showed a better diagnostic performance than T2-weighted images plus contrast-enhanced T1-weighted images in identifying deep myometrial invasion.

In our study, the staging accuracy with MRI was 92.9 per cent for DWI and 85.7 per cent for gadolinium-enhanced T1-weighted images. The depth of myometrial invasion was underestimated in one lesion on DWI and two lesions on gadolinium-enhanced T1-weighted images. Heterogeneous contrast enhancement of the myometrium made it difficult to detect the tumor margins, resulting in underestimation in these patients. Gallego et al. [29] compared the results of conventional MRI sequences, DWI-generated ADC maps, and intraoperative frozen-section in 51 patients with endometrial cancer to detect the degree of myometrial invasion. They reported that ADC maps were superior to conventional sequences in defining the depth of myometrial invasion.

Restricted diffusion in endometrial carcinoma has been reported to be due to the increased cellular density and thus decreased extracellular space and restricted movement of water molecules [24,30]. Therefore, ADC values of high-grade adenocarcinomas with high cellular density are expected to be lower compared to the low-grade adenocarcinomas. Even Rechichi et al. [21] have described that the tumor ADC value did not correlate with histological tumor grade, in agreement with recently published works by Shen et al. [18] and Lin et al. [27]. The current study also did not find any significant relationship between ADC values and tumor grade. On the other hand, Tamai et al. [24] reported significantly lower ADC values in G3 tumors compared to G1 tumors, while Seo et al. [26] found significantly higher ADC values in G1 tumors compared to G2 or G3 tumors. However, due to the significant overlap between different histological tumor grades, accurately estimating the histological grade by using ADC values is somewhat difficult.

In the current study, the difference between the mean ADC value in FIGO stage 1A and FIGO stage 1B lesions was not statistically significant. These findings confirm the results previously reported by Rechichi et al. [21] and Lin et al. [27]. On the other hand, Husby et al. [31] have reported that mean tumor ADC value was significantly lower in tumors with deep myometrial invasion (ADC value of 0.75 × 10^–3^ mm^2^/s) compared with tumors with superficial myometrial invasion (ADC value of 0.85 × 10^–3^ mm^2^/s).

Our study has some limitations. First of all, it has a small sample size, particularly in the group with malignant patients. In the endometrial cancer group, there were only well-differentiated (G1) and moderately differentiated (G2) tumors, and we did not have a poorly differentiated (G3) group. Second, only 1.5T equipment was used in this study. Lastly, the diagnosis was made by D&C or endometrial biopsy in benign lesions rather than hysterectomy.

In conclusion, DWI with quantitative analysis of ADC allows benign endometrial lesions to be differentiated from endometrial cancer. However, no significant association between the ADC values and the depth of myometrial invasion and the histological tumor grade was found. Further study is needed to confirm our findings.
